# Single nucleotide polymorphism (SNP) in the *doublesex* ( *dsx*) gene splice sites and relevance for its alternative splicing in the malaria vector *Anopheles gambiae*

**DOI:** 10.12688/wellcomeopenres.17572.3

**Published:** 2023-02-06

**Authors:** Oswald Djihinto, Helga D.M. Saizonou, Luc S. Djogbenou

**Affiliations:** 1 Tropical Infectious Diseases Research Centre (TIDRC), University of Abomey-Calavi, Abomey-Calavi, 01BP526 Cotonou, Benin; 2 Institut Régional de Santé Publique, University of Abomey-Calavi, Ouidah, BP 384 Ouidah, Benin; 3 Department of Vector Biology, Liverpool School of Tropical Medicine, Pembroke Place, Liverpool L3 5QA, UK

**Keywords:** SNP, alternative splicing, dsx gene, Anopheles gambiae, malaria

## Abstract

**Background:** Malaria burden continues to be significant in tropical regions, and conventional vector control methods are faced with challenges such as insecticide resistance. To overcome these challenges, additional vector control interventions are vital and include modern genetic approaches as well as classical methods like the sterile insect technique (SIT). In the major human malaria vector *Anopheles gambiae*, a candidate gene favourable for sterility induction is the *doublesex* ( *dsx*) gene, involved in mosquitos’ somatic sexually dimorphic traits determination. However, the pathways that trigger the signal of *dsx* gene exon skipping alternative splicing mechanism in anopheline mosquitoes are not well characterized. This study aims to screen the *An. gambiae dsx *gene splice site sequences for single-nucleotide polymorphisms (SNPs) that could be critical to its alternative splicing.

**Methods:** Variant annotation data from Ag1000G project phase 2 was analysed, in order to identify splice-relevant SNPs within acceptor and donor splice sites of the *An. gambiae dsx* gene ( *Agdsx*).

**Results: **SNPs were found in both donor and acceptor sites of the *Agdsx*. No splice-relevant SNPs were identified in the female-specific intron 4 acceptor site and the corresponding region in males. Two SNPs (rs48712947, rs48712962) were found in the female-specific donor site of exon 5. They were not specific to either males or females as the rs48712947 was found in female mosquitoes from Cameroon, and in both males and females from Burkina Faso. In the other splice sites, the intron 3 acceptor site carried the greatest abundance of SNPs.

**Conclusions:** There were no gender association between the identified SNPs and the random distribution of these SNPs in mosquito populations. The SNPs in *Agdsx* splice sites are not critical for the alternative splicing. Other molecular mechanisms should be considered and investigated.

## List of abbreviations

***Agdsx*:** *Anopheles gambiae doublesex* gene

***dsx***: doublesex gene

**ESE**: Exonic Splicing Enhancers

**ESI**: Exonic Splicing Silencers

**Fle:** Femaleness gene

**hnRNPs:** heterogeneous nuclear ribonucleoproteins

**ISE**: Intronic Splicing Enhancers

**ISI**: Intronic Splicing Silencers

**PTMs:** post-translational modifications

**SIT**: Sterile Insect Techniques

**SNP:** Single Nucleotide Polymorphism

***Sxl***: Sex lethal gene

***TRA***: Transformer transcription factor

***TRA2***: Transformer 2 transcription factor

## Introduction

Malaria is a vector-borne infectious disease caused by the protozoan parasite belonging to the *Plasmodium* genus ^1^. The transmission occurs among humans through the bite of the female *Anopheles* mosquito. This disease is among the top ten causes of death in low-income countries ( World Health Organization) ^2^ and continues to take a heavy toll on communities, especially in African regions. The malaria transmission cycle involves four major elements: the host (human), the parasite, the vector, and the environment ^3^. In the absence of effective vaccine or sustainable treatment options, vector control is the cornerstone of malaria management and is based on the prevention of human-host contact and reduction in vector population density ^1, 4^. The traditional vector control strategies rely on long-lasting insecticidal net (LLIN) distribution and indoor residual sprays (IRS) which have contributed to the decreasing malaria cases and mortality ^5, 6^. However, vector resistance against the existing insecticides is increasing in natural mosquito populations ^7– 9^.

The widespread of insecticide resistance in natural vector populations has intensified researches on alternative malaria vectors control strategies. Alternative tools for vector control have included technologies such as cytoplasmic incompatibility with the use of natural *Wolbachia* bacteria infection ^10, 11^; repressible dominant lethal systems in *Aedes aegypti* ^12, 13^; Y-chromosome shredding gene drive (gene drive cassette that also incorporates a programmable endonuclease that shreds the Y chromosome, converting XY males into fertile XO females) ^14^; and the genetic sterilisation of *Anopheles* sp., known as Sterile Insect Techniques (SIT) ^15^. The SIT technique, as firstly developed, is based on the repeated, high-density release of radio-sterilized males, through gamma radiation, into the environment in order to compete with wild males for mating with the native female anopheles mosquitoes, hindering the production of offspring ^16, 17^. Indeed, mated females will not produce viable offspring, resulting in reduced population numbers or even local elimination of the target species. However, instead of exposing males to a source of radiation, sterility could be induced by genetic modification of the mosquito genome and may improve the effectiveness of classical SIT-based approaches ^15^.

In *An. gambiae*, one of the major malaria vectors, population suppression strategies are already under investigation by targeting the gender determination genes such as the *doublesex* ( *dsx*) transcription factor gene ^18, 19^. Therefore, the *Anopheles gambiae doublesex* gene ( *Agdsx*) represents a useful candidate gene for genetic manipulation and improvement of the alternative mosquito control technologies. Interest in this gene comes from the fact that it undergoes alternative splicing and results in female and male-specific transcripts necessary for gender determination in this species ^20^. The use of transgenic tools in anopheline mosquitoes through targeting the *dsx* gene could improve the sterility induction and genetic sexing which are major requirements for genetic SIT technologies. However, the molecular mechanisms underlying gender determination are highly variable.

The only well-known model of the *dsx* splicing comes from the fly *Drosophila melanogaster* sex determination pathway ^21^. The *dsx* gene acts as a transcription factor targeting several genes which have mostly sex- and tissue-specific functions that determine somatic sexual dimorphism traits in later stages of sexual development ^22, 23^. Transformer ( *TRA*) and Transformer 2 ( *TRA2*) are the key regulatory factors of the female-specific alternative splicing of *dsx* pre-mRNA ( *dsxF* isoform) under the control of the Sex lethal gene ( *Sxl*) product while the absence of *TRA* (non-productive form) leads to the male-specific splicing ( *dsxM* isoform) in fruit fly ^22^. Unfortunately, *An. gambiae dsx* gene ( *Agdsx*) has different gene organization and regulatory elements positions suggesting that *Agdsx* gender-specific splicing event is caused by a mechanism different from that of the *D. melanogaster dsx* ^20, 24^. Recently, it was reported that in *An. gambiae*, *femaleness* gene ( *Fle*) is necessary for the splicing of *dsx* into the female-specific mRNA ( *AgdsxF*) ^25^. However, *Fle* is not involved in the *dsx* splicing into the male-specific transcript ( *AgdsxM*) ^25^. Indeed, *Yob1* gene (Y-linked) which is activated at earlier stage of zygotic transcription and expressed all throughout a male's life, regulates male-specific *dsx* splicing ^26^.

**Figure 1.**
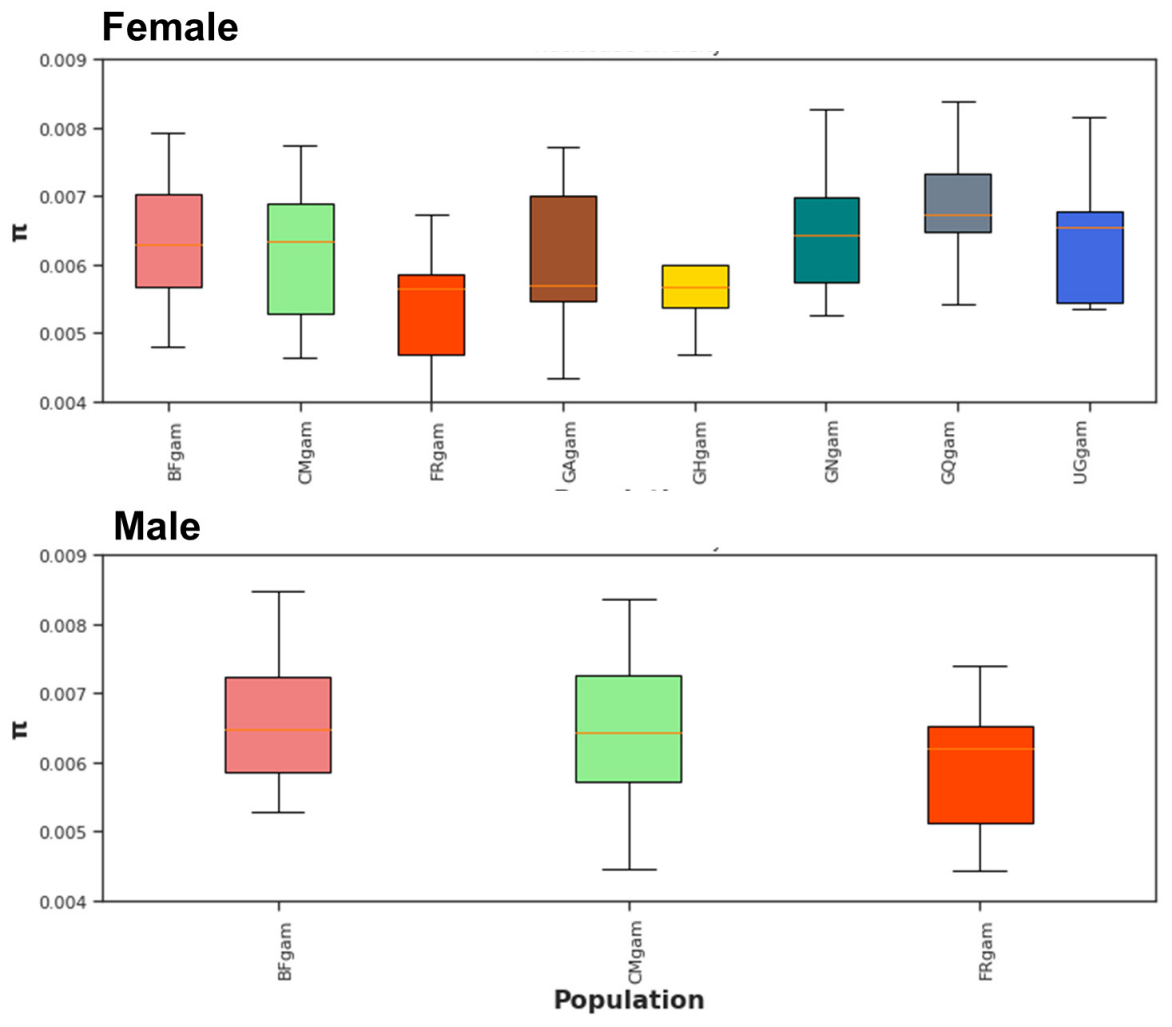
Schematic of common and specific splice sites of *dsx* in *An. gambiae* males and females. Exons are represented irrespective of their size by black boxes. Straight black lines indicate introns. Coloured lines above exon/intron junction indicate donor splice sites. Coloured lines above intron/exon junction indicate acceptor splice sites. Same coloured line above exon/intron or intron/exon boundaries indicate common splice sites in both *AgdsxF* and *AgdsxM* transcripts. The red and green splice sites are specific to *AgdsxF* transcript.

*Agdsx* is located in the 17C band of the chromosome 2R (2R: 48703664 - 48788460) on the reverse strand. The gene is 84.8 kb long and encodes *AgdsxM* and *AgdsxF* transcripts. *AgdsxM* transcript (6975 bp) is shorter than that of *AgdsxF* (8667 bp). The difference between the two gender-specific transcripts is due to the alternative splicing of exon 5. The latter is a cassette exon, which is retained in female and skipped in male transcript. The whole sequence of the female-specific exon 5 is included in the male intron 4 region and is spliced out. This gene structure causes a shift in intron/exon number in male. Thus, although male and female share the same exon/intron or intron/exon boundaries, they have common and specific splice sites ( Figure 1). Though it was demonstrated that *Fle* and *Yob1* genes control respectively *AgdsxF* and *AgdsxM* specific splicing, the pathways triggering the signal of *dsx* gene exon skipping alternative splicing mechanism in *An. gambiae* are not well characterized.

The exon definition by the spliceosome requires interplays between splice sites on either side of the exon. Donor sites (5’-splice site) are defined by GT dinucleotide at the 5’ end of exon-intron border, while AG dinucleotide defined acceptor sites (3’-splice site) at the 3’ end of intron-exon border ^27^. In mammalian cells, the presence of genetic variations such as single nucleotide polymorphisms (SNPs) within the donor and acceptor splice sites is susceptible to influence the splicing and might lead to changes in normal splicing pattern ^28– 30^. The presence of SNP at the acceptor splice site of several genes is reported in human and lead to the alternative splicing of the corresponding genes ^28^. Indeed, in humans, splicing signals are a common point of mutations. Most of the splicing mutations analysed so far directly influence the conventional consensus splicing sequence, and consequently lead to skipping of the adjacent exon ^29^. Lamba *et al*., revealed that a nonsynonymous SNP (15631G>T), which disrupted an exonic splicing enhancer (in exon 4), and a SNP (15582C>T) in an intron-3 branch point are responsible for the skipping of exons 4, 5, and 6 of cytochrome 2B6 ( *CYP2B6*) in females human liver ^31^. Furthermore, coding single-nucleotide polymorphisms (cSNPs) are thought to have the same effect on splicing ^29^. Moreover in animals, especially in cattle, the *ectodysplasin 1* gene ( *ED1*) produces two isoforms that result from alternative splicing. It was reported that this alternative splicing event in *ED1* mRNA is caused by a point mutation found in the 5′ splice donor site of intron 8 ^32^. Gargani *et al.*, have also showed that another single nucleotide polymorphism (SNP) in the exonic splicing enhancer (ESEs) of the exon 8 in *ED1* mRNA leads to the exon skipping in cattle ^33^. In the marine fish *Trachinotus anak*, Fan *et al.*, have identified a single intronic SNP at the first exon/intron boundary of the gender-determining gene *17b-hydroxysteroid dehydrogenase 1* ( *hsd17b1*) ^34^. Specifically, this SNP was demonstrated to affect *hsd17b1* splicing, leading to female development ^34^.

Taking together these observations in humans and animal models, we hypothesized that the same events could be possible in insects and that SNPs could occur in acceptor and/or donor splice sites in mosquitoes that might result in the splice variation. The current report seeks then to screen *Anopheles gambiae doublesex* gene ( *Agdsx*) splice site sequences for single-nucleotide polymorphisms (SNPs) that could be associated with alternative splicing.

## Methods

### Sequence data and mosquito samples

Genomic sequences used in this study came from the Anopheles 1000 genomes (Ag1000G) project phase 2 released in 2017 ^24^. The SNP annotation was downloaded (ag1000g.phase2.ar1.variants.pass.2R.vcf.gz, November 11, 2019) from the Malaria Genomic Epidemiology Network (MalariaGEN) website. This file contain all SNPs identified in mosquito whole genomes and that pass the variant filtering process described by 24. Only *Anopheles gambiae s.s*. samples were considered in our study. These mosquito samples were collected from natural populations from 2002 to 2012 in eight African countries ( Table 1). The reference sequence of *Agdsx* (AGAP004050) was also downloaded from Vectorbase website.

**Table 1.** Sampling locations of *An. gambiae* mosquitoes from the Ag1000G phase 2 project.

Country	Site	Year	Geographic coordinate		Number of species	Number of female	Number of male
			Latitude	Longitude			
Burkina Faso	Bana	2012	11.2330	-4.4720	20	3	17
	Pala	2012	11.1500	-4.2350	46	38	8
	Souroukoudinga	2012	11.2350	-4.5350	26	26	0
Cameroon	Daiguene	2009	4.7770	13.8440	96	81	15
	Gado Badzere	2009	5.7470	14.4420	73	58	15
	Mayos	2009	4.3410	13.5580	105	91	14
	Zembe Borongo	2009	5.7470	14.4420	23	23	0
Equatorial Guinea	Bioko	2002	3.7000	8.7000	9	9	0
France (Mayotte)	Bouyouni	2011	-12.7378	45.1417	1	1	0
	Combani	2011	-12.7787	45.1429	5	2	3
	Karihani Lake	2011	-12.7965	45.1217	3	3	0
	Mont Benara	2011	-12.8570	45.1552	2	1	1
	Mtsamboro Forest Reserve	2011	-12.7027	45.0811	1	1	0
	Mtsanga Charifou	2011	-12.9907	45.1557	8	3	5
	Sada	2011	-12.8521	45.1039	4	1	3
Gabon	Libreville	2000	0.3840	9.4550	69	69	0
Ghana	Madina	2012	5.6685	-0.2193	12	12	0
Guinea	Koraboh	2012	9.2500	-9.9170	22	22	0
	Koundara	2012	8.5000	-9.4170	18	18	0
Uganda	Tororo (Nagongera)	2012	0.7700	34.0260	112	112	0

### Sequence analysis and SNP identification

From the *Agdsx* reference sequence, the list of genomic positions of donor and acceptor sites was extracted. VCFtools version 0.1.15 ( https://vcftools.github.io/index.html) ^35^ was used to extract the SNPs within the genomic region corresponding to the *Agdsx* sequence from the SNPs annotation file. The polymorphic nucleotides were then identified within the splice sequences, in comparison to the reference sequence. SNPs were then visualized using TASSEL version 5.2.63 software ( https://tassel.bitbucket.io/) ^36^. The genomic position of the acceptor sites was used to select SNPs in the last 12 nucleotides of an intron preceding the 3’ splice pattern NYAG and in the first six nucleotides of an exon. In donor splice sites, SNPs were identified within in the last six nucleotides of an exon and the first 16 nucleotides in an intron. The average nucleotide diversity at the *dsx* locus between male and female was calculated using scikit-allel version 1.2.1 ( https://scikit-allel.readthedocs.io/en/stable/) ^37^ in order to determine whether SNPs density at the *dsx* locus differed between the two genders.

## Results

### Identification of *An. gambiae dsx* gene ( *Agdsx*) donor and acceptor splice sites sequence

Male and female mosquitoes share exon 1, 2, 3, 4 and 6 donor splice sites while exon 5 donor site is specific to female as it is only recognized by the spliceosome in females ( Table 2). Similarly, both male and female share intron 1, 2, 3, and 6 acceptor sites. Male intron 4 and female intron 5 share the same 3’ end as the female, and exon 5 is included in the male intron 4 sequence. However, females have the intron 4 specific acceptor site, as the cassette exon 5 definition is not established in males ( Table 2).

**Table 2.** Splice donor and acceptor sites within the double sex ( *dsx*) gene *of Anopheles gambiae*.

Splice donor sites							
Gender	Exon	Size	Exon position		Splice site sequence	Site position	
			Start	End		Start	End
Male/Female	1	1415	48788460	48787046	tatttg/gtaagtaaatatgcaa	48787051	48787030
Male/Female	2	1445	48785629	48784185	TGGGAG/gtaagtacgatcatgc	48784190	48784169
Male/Female	3	45	48747737	48747693	TACCTG/gtaagtaaatataatt	48747698	48747677
Male/Female	4	135	48715295	48715161	ACGAAG/gtaagctggcgatgat	48715166	48715145
Female	5	1692	48714648	48712957	cagaag/gtatggtaagacggcc	48712962	48712941
Male/Female	6	1267	48712794	48711528	aaaaag/gtaagtgtgggtagta	48711533	48711512
Male/Female	7	2668	48706331	48703664	None		
Splice acceptor sites							
Gender	Intron	Size	Intron position		Splice site sequence	Site position	
			Start	End		Start	End
Male/Female	1	1416	48787045	48785630	gtacgtttgattgcag/atctcc	48785645	48785624
Male/Female	2	36447	48784184	48747738	ttgctctccttttcag/CTACTC	48747753	48747732
Male/Female	3	32397	48747692	48715296	ttccgccccgtttcag/ACGACG	48715311	48715290
Female	4	512	48715160	48714649	tttatgtttaacacag/GTCAAG	48714664	48714643
Male	4	2366	48715160	48712795	tgtaacccccaaaaag/gtaaac	48712810	48712789
Female	5	162	48712956	48712795			
Male/Female	6	5196	48711527	48706332	cgcttcctcaaaatag/atcgat	48706347	48706326

Splice site sequences are given in 5’➔3’ direction on the reverse strand. Exonic coding sequences are shown in uppercase letters, and non-coding regions are in lowercase letters. The 12 bp preceding the 3’ splice-acceptor site (NYag) is indicated, where Y = T or C and N = any nucleotide.

### SNPs in female-specific intron 4 acceptor and exon 5 donor splice sites

**Figure 2.**
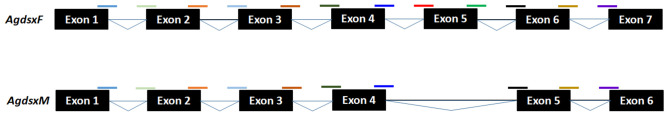
Nucleotide diversity (π) at *Agdsx* locus within mosquito populations. BF: Burkina Faso; CM: Cameroon; FR: Mayotte; GA: Gabon; CH: Ghana; GN: Guinea; GQ: Equatorial Guinea; UG: Uganda. No differences in nucleotide diversity were observed within male or female mosquito populations.

Along the *Agdsx* gene, 17,196 polymorphic sites were identified. Wherever both male and female mosquitoes are present (in Burkina Faso, Cameroon and Mayotte), the nucleotide diversity is similar between both genders ( Figure 2). This was expected as male and female in each country make up a single population. In addition, no difference in the nucleotide diversity was observed between male populations from the three countries (Burkina Faso, Cameroon and Mayotte) ( Figure 2, top panel). The same trend was observed between female populations as well (Burkina Faso, Cameroon, Mayotte, Gabon, Ghana, Guinea, Equatorial Guinea and Uganda).

**Figure 3.**
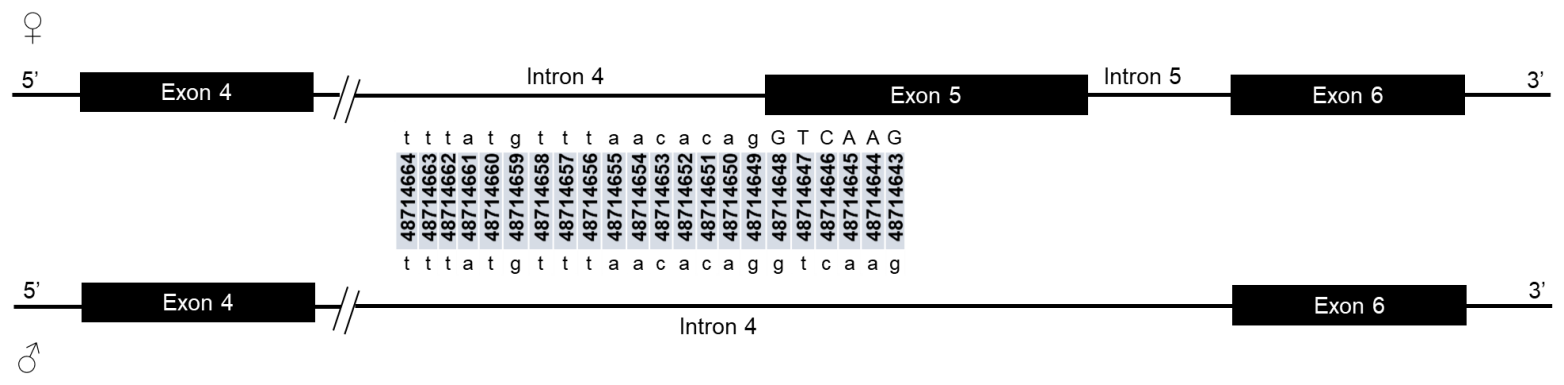
Female-specific intron 4/exon 5 junction and the corresponding region in male. Intron 4 splice acceptor sequence is indicated in female with the genomic positions of each nucleotide. Female-specific exon 5 is included in male intron 4 sequence. The corresponding region of the female intron 4 acceptor site is indicated within male intron 4. Exonic coding sequences are shown in uppercase letters, and non-coding regions are in lowercase letters. No SNP was neither found in the female intron 4 splice acceptor site or in the corresponding region in male.

**Figure 4.**
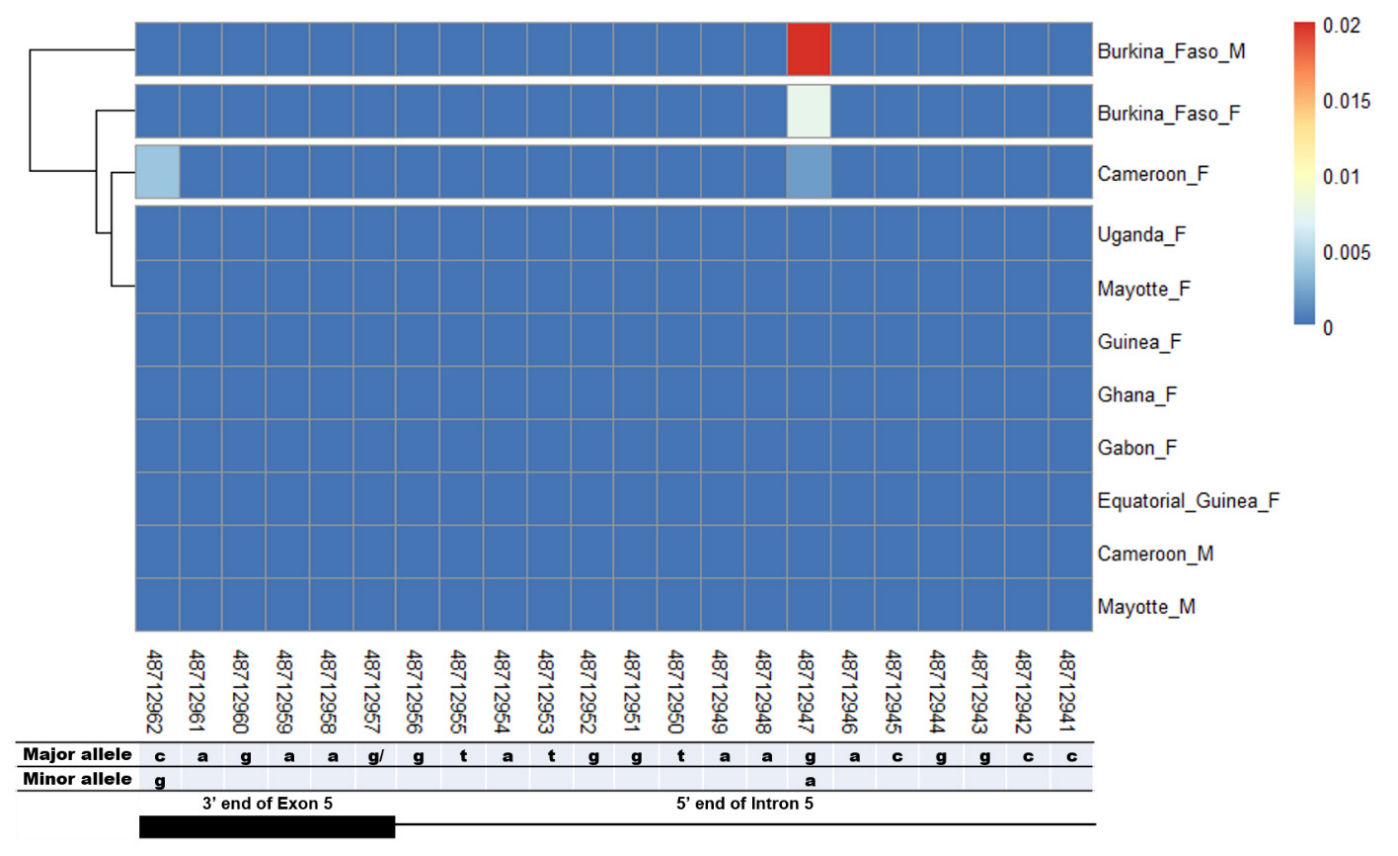
SNP within female specific exon 5 donor splice site and the corresponding region in male. The coloured and dark blue squares denote respectively the presence or absence of SNP. Each row represents male ( **M**) or female ( **F**) mosquito population. At the bottom, the numbers are the genomic positions of each nucleotide. The major and minor allele (where applicable) are indicated for each nucleotide position. The key colour is scaled to the minor allele frequency. The black box and the black line respectively depict an exonic and intronic regions covered by the splice site in females. The corresponding region of this female donor splice site within male intron 4 was analysed. SNPs were found at very low frequencies at position 48,712,947 in Burkina Faso and Cameroon female and Burkina Faso male populations. SNP at 48,712,962 was found only in Cameroon female population. No gender-specific SNP was identified.

The potential splice-relevant SNPs that could trigger the female-specific exon 5 skipping should be in the intron 4 acceptor and exon 5 donor sites. However, there was no SNP in the acceptor sequence of female-specific intron 4 nor in the corresponding male region ( Figure 3). However in the female-specific exon 5 donor site, two SNPs (rs48712947, rs48712962) were found. Nevertheless, they were not specific to females as the rs48712947 was found in Cameroon female mosquitoes and in both males and females from Burkina Faso ( Figure 4). The rs48712962 was absent in the male mosquito population, while it was found only in females in Cameroon. The minor allele frequencies (MAF) of both SNPs identified were very low in each population. The MAF of rs48712947 and rs48712962 amounted to less than 1% in each female population, and only 2% of Burkina Faso male carried the rs48712947.

### SNPs in other splice sites of Agdsx

**Figure 5.**
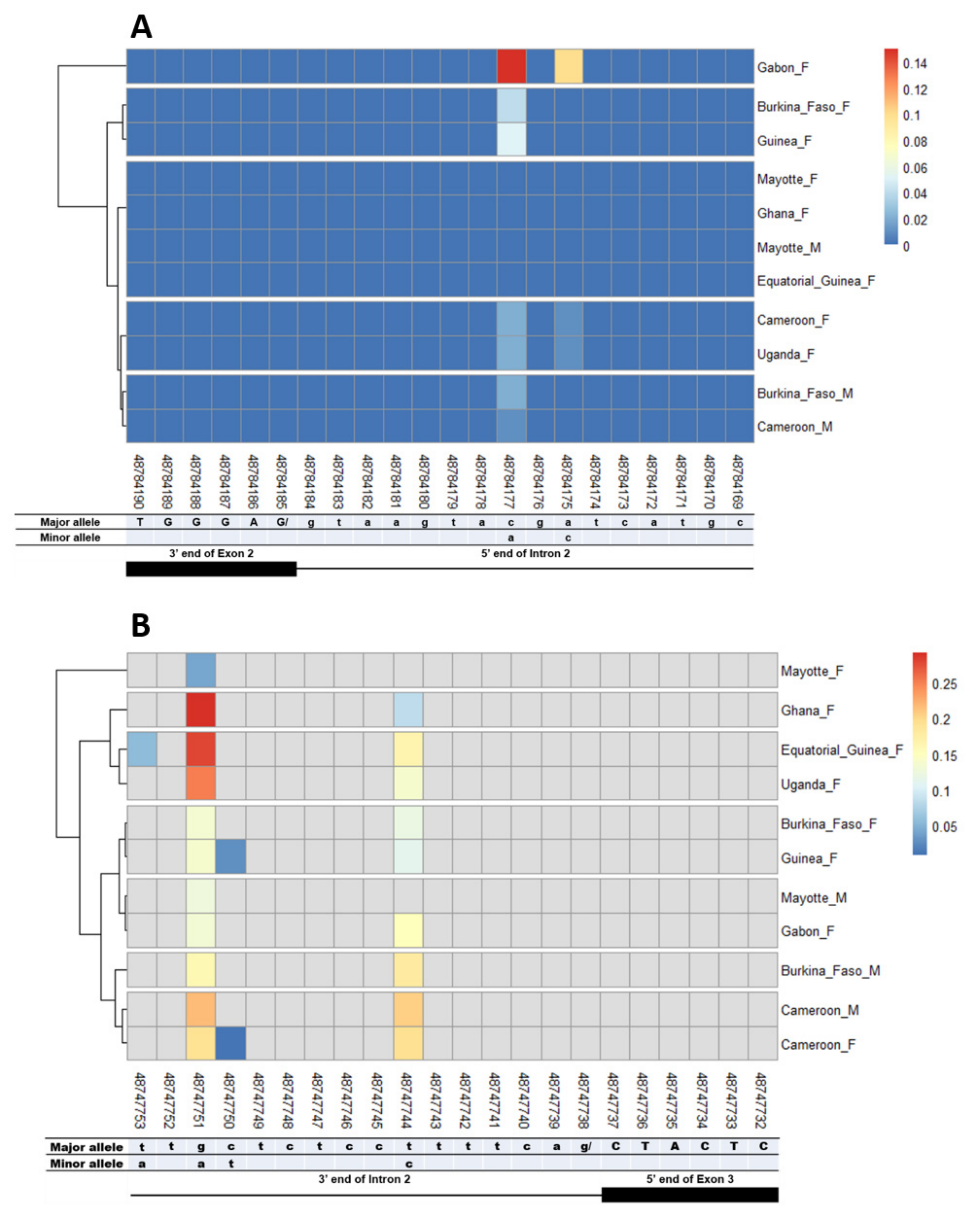
Single nucleotide polymorphism within exon 2 and intron 2 splice sites. **A**) SNPs within exon 2 donor splice site between male and female *An. gambiae* mosquitoes. Each row represents male ( **M**) or female ( **F**) mosquito population. At the bottom, the numbers are the genomic positions of each nucleotide. The major and minor allele (where applicable) are indicated for each nucleotide position. The key colour is scaled to the minor allele frequency. The black box and the black line respectively depict an exonic and intronic regions covered by the splice site in females. The uppercase and lowercase letters denote respectively coding and non-coding region. **B**) SNPs within intron 2 acceptor splice site between male and female *An. gambiae* mosquitoes. The uppercase and lowercase letters denote respectively coding and non-coding region.

**Figure 6.**
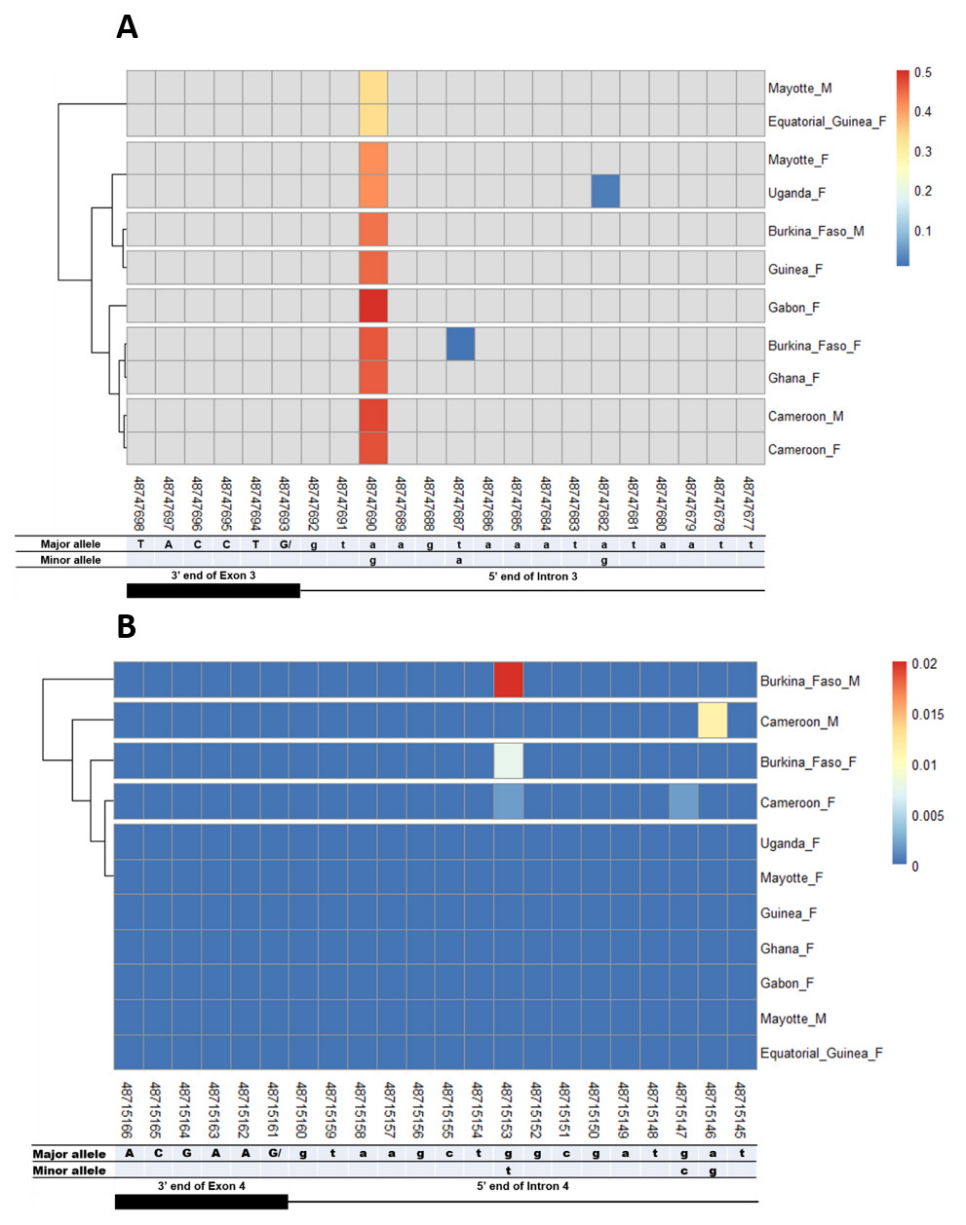
Single nucleotide polymorphism within exon 3 and exon 4 splice sites. **A**) SNPs within exon 3 donor splice site between male and female *An. gambiae* mosquitoes. Each row represents male ( **M**) or female ( **F**) mosquito population. At the bottom, the numbers are the genomic positions of each nucleotide. The major and minor allele (where applicable) are indicated for each nucleotide position. The key colour is scaled to the minor allele frequency. The black box and the black line respectively depict an exonic and intronic regions covered by the splice site in females. The uppercase and lowercase letters denote respectively coding and non-coding region. **B**) SNPs within exon 4 donor splice site between male and female *An. gambiae* mosquitoes. The uppercase and lowercase letters denote respectively coding and non-coding region.

**Figure 7.**
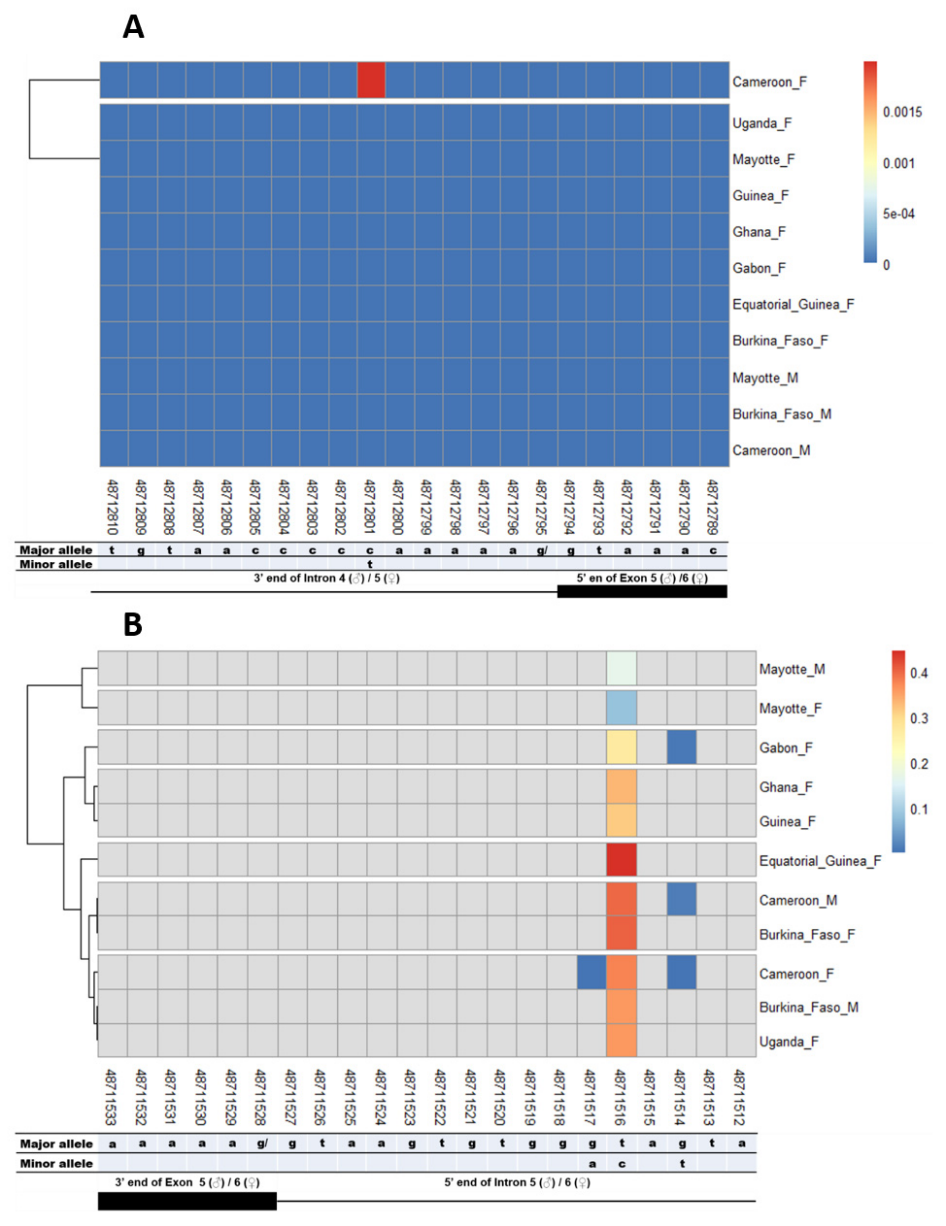
Single nucleotide polymorphism in the last *Agdsx* acceptor and donor splice sites. **A**) SNPs within the common acceptor site (Intron 4/5) between male and female *An. gambiae* mosquitoes. The female (♀) specific exon 5 is included in the male (♂) intron 4 sequence making a shift in exon number is male. Thus, the male intron 4 and female intron 5 share the same 3’ end. Similarly, the male exon 5 and female exon 6 share the same 5’ end region. **B**) SNPs within the common donor site (Exon 5/6) between male and female *An. gambiae* mosquitoes. The female (♀) specific exon 5 is included in the male (♂) intron 4 sequence making a shift in exon number is male. Thus, the male exon 5 and female exon 6 share the same 3’ end. Similarly, the male intron 5 and female intron 6 share the same 5’ end region.

**Figure 8.**
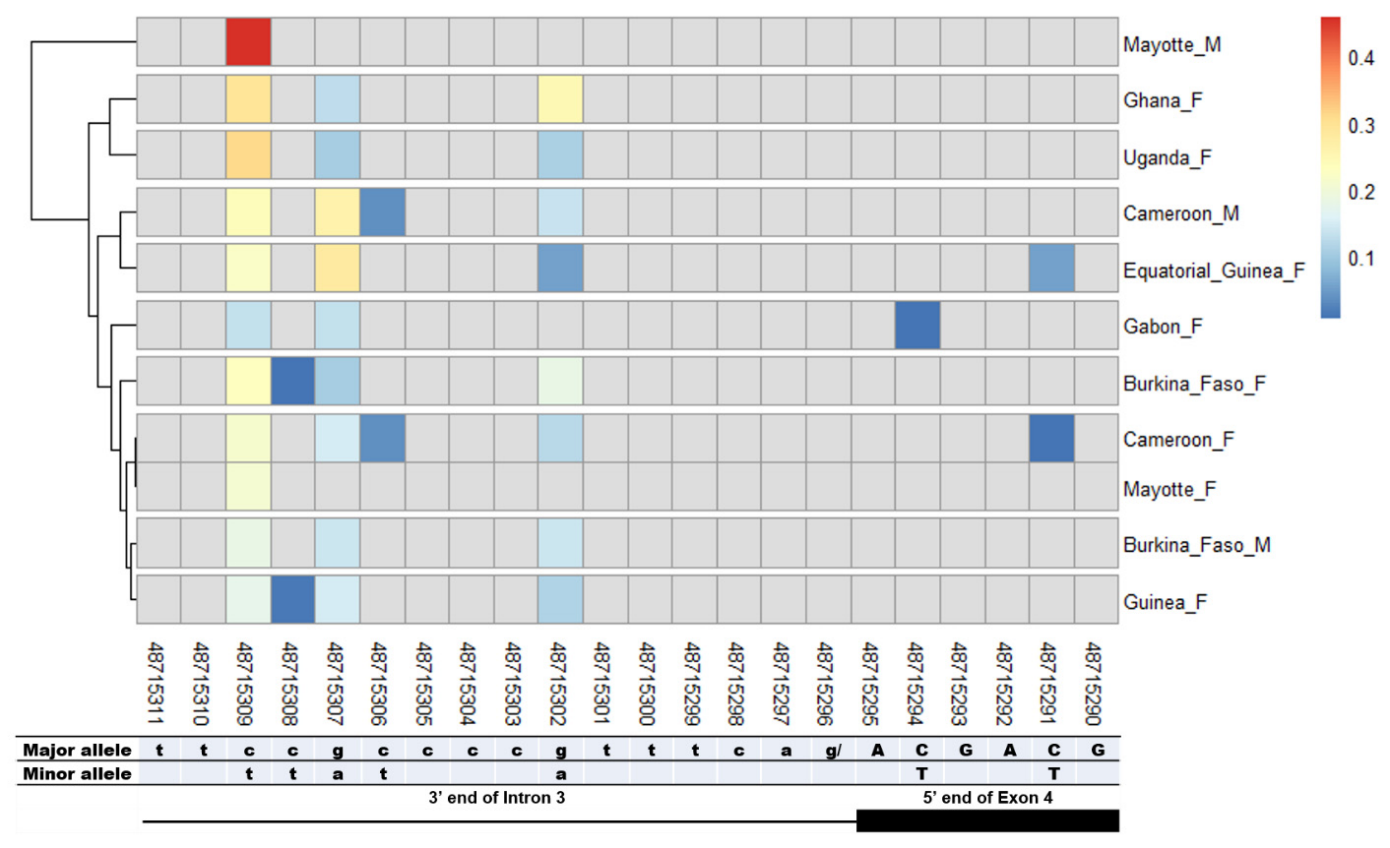
SNP within intron 3 acceptor splice site between *An. gambiae* male and female mosquitoes. The uppercase and lowercase letters denote respectively coding and non-coding region. The coloured and grey squares denote respectively the presence or absence of SNP. Each row represents male ( **M**) or female ( **F**) mosquito population. At the bottom, the numbers are the genomic positions of each nucleotide. The major and minor allele (where applicable) are indicated for each nucleotide position. The key colour is scaled to the minor allele frequency. The black box and the black line depict respectively an exonic and intronic regions covered by the splice site in each mosquito gender. No gender-specific SNP was identified.

The other splice sites were also examined for identification of gender-specific SNPs. No SNP was found in the shared exon 1 donor, introns 1. No splice-relevant SNP was found in the other donor ( Figure 5A, Figure 6, and Figure 7B) and acceptor ( Figure 5B, and Figure 7A) splice sites. The highest number of SNPs (7) was found in the common intron 3 acceptor site sequence (rs48715291, rs48715294, rs48715302, rs48715306, rs48715307, rs48715308, rs48715309) ( Figure 8). However, each of these SNPs occurred in a non-specific manner in both male and female populations, with variable minor allele frequencies.

## Discussion

The *An. gambiae doublesex* ( *Agdsx*) gene is a candidate gene of interest for genetic SIT strategy ^18, 19, 25^. The translation and the success of using *dsx* in SIT methodology require a clearer understanding of the genetic bases of the gender determination pathway. This study screened the *Agdsx* donor and acceptor splice sites for identification of splice-relevant SNPs.

The alternative splicing of *Agdsx* gene is governed by exon 5 skipping in male mosquitoes ^20^ suggesting a silencing mechanism of the female-specific splice sites recognition (intron 4 acceptor and exon 5 donor sites) by the splicing machinery in males. Such silencing mechanism could be due to changes in splice site sequence. However, female-specific intron 4 acceptor site sequence is present within male intron 4 and no SNP was found in this sequence in both males and females. The SNPs rs48712947 and rs48712962 identified in female-specific exon 5 donor site were neither splice-relevant nor gender-specific. They appeared only is two mosquito populations (Burkina Faso and Cameroon) over the eight populations considered. In each population where these SNPs have been identified, they appeared in very few individuals, less than 1% in females and no more than 2% in males. These observations suggest that the *Agdsx* cassette exon 5 was not associated with changes in splice site patterns due to the presence of SNPs. The presence of SNPs in the other splice sites had also different distribution and were non-specific to the gender of the mosquitoes.

Another factor for exon skipping is the pyrimidine content of the polypyrimidine tract in acceptor splice sequence. Indeed a poor polypyrimidine tract causes a shift of the splicing machinery to the next acceptor site, leading to the skipping as the case of exon 4 skipping in male *Drosophila* ^21^. In *Anopheles gambiae* the number of pyrimidine (8) in the 12bp preceding the acceptor site pattern (acag) ( Table 2) in the female-specific intron 4 is the same in the male corresponding region. The same number of pyrimidines in this acceptor sequence was reported by Scali *et al.* ^20^. Furthermore, the authors found that this number did not differ from the consensus number of pyrimidines (8.69) in *An. gambiae* splice acceptor sites, and concluded that the intron 4 site may not be a weak acceptor site ^20^. Overall, these findings add further evidence that other mechanisms underlie the alternative splicing in *An. gambiae* and open perspective for further investigation on the molecular mechanisms of *Agdsx* splicing.

It was known that the regulation of alternative splicing evolved tans‐acting splicing factors, such as serine-arginine-rich (SR) family proteins and heterogeneous nuclear ribonucleoproteins (hnRNPs) that bind to the auxiliary silencer and enhancer *cis*-element (ESE: exonic splicing enhancers; ESI: exonic splicing silencers; ISE: intronic splicing enhancers; ISI: intronic splicing silencers) ^38– 40^. Similar regulatory *cis*-elements were found in *Drosophila melanogaster* female-specific exon and putative homologs were identified in *An. gambiae* female-specific exon 5 ^20^. Therefore, further molecular analyses are needed toward characterizing these regulatory sequence and their binding trans-factors in order to underpin the somatic sex determination in *An. gambiae*.

Moreover, the epigenetic system was also reported to regulate the alternative splicing in mammalian and other insect cells. Indeed, it was demonstrated that changes in DNA cytosine methylation on the gene body in honey bees may lead to alternative splicing ^41– 43^. Also histone post-translational modifications (PTMs) such as lysine acetylation and methylation were associated to the alternative splicing event ^44– 46^. Consequently, similar mechanisms could happen in the malaria vector *An. gambiae* to regulate gene alternative splicing. However, no significant DNA methylation was reported in Diptera including *An. gambiae* ^47, 48^. Then, the only epigenetic modifications that could be linked to the alternative splicing in this species are histone PTMs. Indeed, the methylation and acetylation of lysines 4, 9 and 29 of histone H3 were reported in *An. gambiae* ^49, 50^. Then, it will be interesting to evaluate whether such histone modifications enrichment in *Agdsx* between male and female mosquitoes could be critical for *dsx* alternative splicing.

## Conclusion

Sustainable vector control strategies will rely on the integrated use of chemical and biological vector control. Given the potential of the *Agdsx* gene for SIT, the understanding of mechanisms of it regulation could help to improve the tools engineering targeting this locus. SNPs were identified within the *Agdsx* and their putative association with the *dsx* alternative splicing was analysed. No splice-relevant SNP was found in the specific male and female splice site. The SNPs were distributed in few proportion of individuals in the populations where they were identified. With the advances in genetic biotechnology, other mechanisms remain to be explored for providing solid background on somatic sexual fate determination in *Anopheles gambiae*. This will pave the way to find new biochemical and genetics target for malaria vector control.

## Data Availability

Figshare: Data underlying Single nucleotide polymorphism (SNP) in the doublesex (dsx) gene splice sites and relevance for its alternative splicing in the malaria vector *Anopheles gambiae*. https://doi.org/10.6084/m9.figshare.18589781.v1 This project contains the following underlying data: **Dsx_f.h5**. (Data used to plot nucleotide diversity in female populations) **Dsx_f.h5**. (Data used to plot nucleotide diversity in male populations) **dsx_variant_seq_norm_F**. (VCF format dataset containing SNPs in *doubesex* gene sequence of female mosquitoes) **dsx_variant_seq_norm_M**. (VCF format dataset containing SNPs in *doubesex* gene sequence of male mosquitoes) **Female_sample_ID**. (Dataset of the accession numbers of females sequences in the dsx_variant_seq_norm_F file) **Female_sample_ID**. (Dataset of the accession numbers of males sequences in the dsx_variant_seq_norm_M file). Data are available under the terms of the Creative Commons Zero "No rights reserved" data waiver (CC0 1.0 Public domain dedication).
