# Supplementary material for: Single nucleotide polymorphism (SNP) in the doublesex ( dsx) gene splice sites and relevance for its alternative splicing in the malaria vector Anopheles gambiae
Source: Wellcome Open Res. 2023 Feb 6;7:31. Originally published 2022 Jan 28. [Version 3] doi: 10.12688/wellcomeopenres.17572.3 (PMC10397894; doi:10.12688/wellcomeopenres.17572.3)
Supplement: Peer review history

# Peer review: Reviewer response for version 3

**DOI:** 10.21956/wellcomeopenres.21053.r58870

**Published:** 2023-08-02

**Authors:** Moussa W Guelbeogo

**Affiliations:** 1 Centre National de Recherche et Formation sur le Paludisme (CNRFP), Ouagadougou, Burkina Faso

**Review comments**

**General comments**

 The authors of this manuscript have addressed a topic issue relating to the need for new vector control tools in regard of the declining of effectiveness of current insecticide-based tools. They are looking how they can increase scientific finding to improve SIT tools to tackle the major vector An gambiae. They aimed to explore the pathways that trigger the signal of dsx gene exon skipping alternative splicing mechanism in anopheline mosquitoes. They screen the An. gambiae dsx gene splice site sequences for single nucleotide polymorphisms (SNPs) that could be critical to its alternative splicing.

 The present version of the manuscript is well improved, and the results are well presented. The content fits very well to the title. However, it is remaining minor corrections to fix.

**Introduction**

 1. ‘’This disease is among the top ten causes of death in low-income countries (World Health Organization)2 and continues to take a heavy toll on communities, especially in African regions’’. Remove the ‘’World Health Organization’’ keep only the same type of citation

**Methodology**

 Very suitable to address the research question.

 Line’’ This file contain all SNPs identified in mosquito whole genomes and that pass the variant filtering process described by 24’’ I suggest to add Clarkson CS and colleague and then put the citation number 24

 Table1: Head of column 6 : the change the sentence ‘’ Number of species’’ by ‘Number of specimens’’  the species is An gambiae

**Results**

 Figure 1 : Please, can you explain or give the meaning of BFgam, CMgam, FRgam, GAgam, GHgam, GNgam, GQgam and UGgam? it can help to do the connection with the study countries.

 Figure2 :  Check the sentence in the title: ‘’BF: Burkina Faso; CM: Cameroon; FR: Mayotte; GA: Gabon; CH: Ghana; GN: Guinea; GQ: Equatorial Guinea; UG: Uganda’’. I do not understand why this description here, because I don't see any connection between this description and the figure. The description must be deleted.

**Discussion**

 It looks fine.

**Conclusion**

 Why the *An coluzzi* was not included in the study. The result of your study has opened some interesting questions to address these question, but you must include the *An coluzzi* for the next studies because it is one the major vector and widely distributed in western Africa

Is the work clearly and accurately presented and does it cite the current literature?

Yes

If applicable, is the statistical analysis and its interpretation appropriate?

Not applicable

Are all the source data underlying the results available to ensure full reproducibility?

Yes

Is the study design appropriate and is the work technically sound?

Yes

Are the conclusions drawn adequately supported by the results?

Yes

Are sufficient details of methods and analysis provided to allow replication by others?

Yes

Reviewer Expertise:

Vector bilogist, genetic  population of vector

I confirm that I have read this submission and believe that I have an appropriate level of expertise to confirm that it is of an acceptable scientific standard.

# Peer review: Reviewer response for version 3

**DOI:** 10.21956/wellcomeopenres.21053.r54729

**Published:** 2023-02-06

**Authors:** Yoosook Lee

**Affiliations:** 1 Florida Medical Entomology Laboratory, Department of Entomology and Nematology, Institute of Food and Agricultural Sciences, University of Florida, Vero Beach, FL, USA

No further edits needed

Is the work clearly and accurately presented and does it cite the current literature?

No

If applicable, is the statistical analysis and its interpretation appropriate?

Partly

Are all the source data underlying the results available to ensure full reproducibility?

Yes

Is the study design appropriate and is the work technically sound?

Yes

Are the conclusions drawn adequately supported by the results?

Partly

Are sufficient details of methods and analysis provided to allow replication by others?

Yes

Reviewer Expertise:

NA

I confirm that I have read this submission and believe that I have an appropriate level of expertise to confirm that it is of an acceptable scientific standard.

# Peer review: Reviewer response for version 2

**DOI:** 10.21956/wellcomeopenres.20704.r53754

**Published:** 2023-01-27

**Authors:** Yoosook Lee

**Affiliations:** 1 Florida Medical Entomology Laboratory, Department of Entomology and Nematology, Institute of Food and Agricultural Sciences, University of Florida, Vero Beach, FL, USA

The paper could be set to ‘approved’ status for me.

 There are some minor edits needed though:

- “ *Moreover, the epigenetic system was also reported to regulate the alternative splicing in mammalian and other insects cells.”* -> change to ‘other insect cells’.
- Some citations (e.g. World Health Organization fact sheet) seems like they should be in a format consistent with other references (hyper link text to numbers).

Is the work clearly and accurately presented and does it cite the current literature?

No

If applicable, is the statistical analysis and its interpretation appropriate?

Partly

Are all the source data underlying the results available to ensure full reproducibility?

Yes

Is the study design appropriate and is the work technically sound?

Yes

Are the conclusions drawn adequately supported by the results?

Partly

Are sufficient details of methods and analysis provided to allow replication by others?

Yes

Reviewer Expertise:

NA

I confirm that I have read this submission and believe that I have an appropriate level of expertise to confirm that it is of an acceptable scientific standard.

# Peer review: Reviewer response for version 1

**DOI:** 10.21956/wellcomeopenres.19431.r51402

**Published:** 2022-07-08

**Authors:** Yoosook Lee

**Affiliations:** 1 Florida Medical Entomology Laboratory, Department of Entomology and Nematology, Institute of Food and Agricultural Sciences, University of Florida, Vero Beach, FL, USA

**Overall comment:**

 The authors should update the Introduction section to reflect the latest development of sex determination research on *Anopheles gambiae *and related species/genes and set reasonable expectations and hypotheses.

 The detailed suggested edits are noted below:

- “ *The malaria transmission cycle involves, four major elements: the host (human), the parasite, the vector and the environment*.”
  Use oxford style commas. “… the vector, and the environment.”
- “ *In the last decade, scientific advances in additional tools for vector control have included technologies such as* …” -> the papers cited for novel vector control tools are over 10 years old. There are newer publications in this realm and should be changed to newer citations or change wording to “In the last two decades, …”
- “The latter technique, SIT, is based on …” -> construct of listing more than two things and referencing ‘the latter’ for the last element is a bit awkward.
- The 2 ^nd^ paragraph of the introduction needs to be changed. As far as I am concerned there has not been radio-sterilized male SIT for Anopheles species although some people have attempted to test it in lab conditions and prepare for field trials. The paper 13 cited for SIT is introducing a genetic tool for inducing SIT not radiation. So explaining general radiation-based SIT method and citing paper 13 is not appropriate. It is especially concerning as this paper pertains to the *Anopheles gambiae* in Africa. Combined with more-than-a-decade-old citations for the alternative vector control strategies, it is somewhat questionable if the authors have due diligence in background research.
- “The use of transgenic tools in anopheline mosquitoes through targeting the *dsx* gene could improve the sterility induction and genetic sexing which are major requirements for SIT technologies.” -> add ‘genetic’ in front of ‘SIT technologies’ as radiation-based SIT does not require genetic sexing.
- The manuscript has mixed use of gender and sex. I suggest using the same and consistent terminology throughout the manuscript.
- “ *Unfortunately, An. gambiae dsx gene (Agdsx) has a different structure suggesting that Agdsx sex-specific splicing event is caused by a mechanism different from that of the D. melanogaster dsx” -> *word ‘structure’ in this case is not clear to readers. Structure is a very generic term and can mean very different things (e.g. RNA structure? Protein structure?). Clarification would be helpful for readers.
- *“Indeed, in humans, splicing signals are a common point of mutations…. Taking together these observations in humans and animal models, we hypothesized that the same events could be possible in insects and that SNPs could occur in acceptor and/or donor splice sites in mosquitoes that might result in the splice variation.*”-> There is some logical gap about expecting mutations in splicing regions determining sex locus. It seems like this is such a fundamental biological function that has huge implications in the downstream process so this region would be likely conserved. The authors should find examples of not just having mutations in splicing regions but splicing mutations in sex-determining genes to be relevant and set reasonable hypotheses/expectations.
- Table 1 – Since the study examined the allele frequency between males and females, Table 1 should provide the number of male and female sequences used for the analysis. Some sites of the Ag1000 data only contain females so generalizing the sex-dependent patterns with skewed data can be misleading.
- “ *Only Anopheles gambiae samples were considered in our study*.” -> Anopheles gambiae s.s. (S form) only? Since the species *gambiae *and *coluzzii* were separated only in 2014, I think it would be best to clarify what you included for the readers.
- The first paragraph of the results section should be in the introduction. Also, readers who are not familiar with the sex determination process would wonder why sex determining gene is located on an autosome, not a sex chromosome. To educate readers, the authors should briefly describe how doublesex on autosome is involved in sex determination of *Anopheles gambiae* based on currently published literature in the introduction.
- Figure 1. It would be best if female and male from the same location is side by side for a direct comparison. Males and females separate with different y-axis scales makes it difficult to compare. FR and GA colors are too similar.
- “ *The An. gambiae doublesex (Agdsx) gene is a candidate gene of interest for SIT, as a candidate for genetic modifications*”-> candidate is repeated twice. Perhaps change it to “… is a candidate gene for genetic SIT strategy” or something to that effect?
- Important paper on this topic like https://www.ncbi.nlm.nih.gov/pmc/articles/PMC7955153/ ^1^ is not cited in this paper.
- “ *According to the D. melanogaster model ^18^, the alternative splicing of Agdsx gene is governed by exon 5 skipping in male mosquitoes ^16^ suggesting a silencing mechanism of the female-specific splice sites recognition (intron 4 acceptor and exon 5 donor sites) by the splicing machinery in males*.” -> I think there is a new paper on this topic from *Anopheles gambiae* which sequenced female and male transcriptome so the author doesn’t have to deduce from the Drosophila sex termination model. Please check the latest papers by Krzywinski for updated literature on *Anopheles gambiae *sex determination.
- “ *They appeared only is two mosquito populations (Burkina Faso and Cameroon) over the eight populations considered.*” -> Burkina Faso and Cameroon are far apart so I wouldn’t minimize how widespread this mutation is based on the number of countries examined (2 out of 8 is still not a low number).

Is the work clearly and accurately presented and does it cite the current literature?

No

If applicable, is the statistical analysis and its interpretation appropriate?

Partly

Are all the source data underlying the results available to ensure full reproducibility?

Yes

Is the study design appropriate and is the work technically sound?

Yes

Are the conclusions drawn adequately supported by the results?

Partly

Are sufficient details of methods and analysis provided to allow replication by others?

Yes

Reviewer Expertise:

Mosquito population genomics

I confirm that I have read this submission and believe that I have an appropriate level of expertise to state that I do not consider it to be of an acceptable scientific standard, for reasons outlined above.

## References

[1] : femaleless Controls Sex Determination and Dosage Compensation Pathways in Females of Anopheles Mosquitoes. *Current Biology* .2021;31(5) : 10.1016/j.cub.2020.12.014 1084-1091.e4 10.1016/j.cub.2020.12.014 PMC795515333417880

# Response

**Published:** 2022-12-03

**Authors:** Oswald Djihinto

**Affiliations:** Tropical Infectious Diseases Research Centre (TIDRC), University of Abomey-Calavi, Benin

**#Reviewer 2. **The authors should update the Introduction section to reflect the latest development of sex determination research on Anopheles gambiae and related species/genes and set reasonable expectations and hypotheses.

***Reply*** *: An attempt was made to update the Introduction.*

**#Reviewer 2.** “The malaria transmission cycle involves, four major elements: the host (human), the parasite, the vector and the environment.” Use oxford style commas. “… the vector, and the environment.”

***Reply*** *: Done*

**#Reviewer 2.** “In the last decade, scientific advances in additional tools for vector control have included technologies such as …” -> the papers cited for novel vector control tools are over 10 years old. There are newer publications in this realm and should be changed to newer citations or change wording to “In the last two decades, …”

***Reply*** *: The sentence has been changed. Please, see lines 66-67.*

**#Reviewer 2.** “The latter technique, SIT, is based on …” -> construct of listing more than two things and referencing ‘the latter’ for the last element is a bit awkward.

***Reply*** *: The sentence was changed. Please see lines 72 in the revised manuscript.*

**#Reviewer 2.** The 2nd paragraph of the introduction needs to be changed. As far as I am concerned there has not been radio-sterilized male SIT for Anopheles species although some people have attempted to test it in lab conditions and prepare for field trials.

***Reply*** *: The second paragraph was changed. We did not mean that there has been radio-sterilized male SIT for Anopheles species. We did mention “the genetic sterilisation of Anopheles species” ( **line 72**). However, we were explaining the principle of SIT approach as firstly developed based on radiation. Please, see lines 72-75.*

**#Reviewer 2.** The paper 13 cited for SIT is introducing a genetic tool for inducing SIT not radiation. So explaining general radiation-based SIT method and citing paper 13 is not appropriate. It is especially concerning as this paper pertains to the Anopheles gambiae in Africa. Combined with more-than-a-decade-old citations for the alternative vector control strategies, it is somewhat questionable if the authors have due diligence in background research.

***Reply*** *: The paper 13 was changed were the general radiation-based SIT method is explained. The alternative vector control strategies are based on tools that have been developed more than decade ago. This justifies the date of the citations used.*

**#Reviewer 2**. “The use of transgenic tools in anopheline mosquitoes through targeting the dsx gene could improve the sterility induction and genetic sexing which are major requirements for SIT technologies.” -> add ‘genetic’ in front of ‘SIT technologies’ as radiation-based SIT does not require genetic sexing.

***Reply*** *: Done*

**#Reviewer 2**. *The manuscript has mixed use of gender and sex. I suggest using the same and consistent terminology throughout the manuscript.*

***Reply*** *: Done*

**#Reviewer 2**. “Unfortunately, An. gambiae dsx gene (Agdsx) has a different structure suggesting that Agdsx sex-specific splicing event is caused by a mechanism different from that of the D. melanogaster dsx” -> word ‘structure’ in this case is not clear to readers. Structure is a very generic term and can mean very different things (e.g. RNA structure? Protein structure?). Clarification would be helpful for readers.

***Reply*** *: Clarification was made as follows: “* *Unfortunately, An. gambiae dsx gene (Agdsx) has different gene organization and regulatory elements positions…”*

*Indeed, Scali et al., (2005) have reported that D. melanogaster female dsx gene has 4 exons while An. gambiae (Agdsx) has 7 exons. The exon 4 is splice out in female Dmdsx transcript while the exon 5 is the cassette exon in Agdsx.* *In *Dmdsx*, the inclusion or the exclusion of the female-specific exon depends on the presence of a weak 3’ splice site in the preceding intron. Genomic sequence and transcript analysis indicate that in *An. gambiae*, splicing into sex-specific transcripts does not depend on the choice of an alternative 3’ splice site (Scali et al., 2005). The 3’ acceptor site preceding exon 5 does not appear to be weak. Although regulatory elements (splice enhancers and potential purine-rich enhancers) are found in the 3’ UTR of exon 5 in Agdsx, these elements are found much further downstream from the 3′ splice acceptor site than in *Drosophila*, immediately upstream of the 5′ donor site of intron 5.*

*Scali C, Catteruccia F, Li Q, Crisanti A. Identification of sex-specific transcripts of the Anopheles gambiae doublesex gene. J Exp Biol. 2005;208: 3701–3709. doi:10.1242/jeb.01819*

**#Reviewer 2**. “Indeed, in humans, splicing signals are a common point of mutations…. Taking together these observations in humans and animal models, we hypothesized that the same events could be possible in insects and that SNPs could occur in acceptor and/or donor splice sites in mosquitoes that might result in the splice variation.”-> There is some logical gap about expecting mutations in splicing regions determining sex locus. It seems like this is such a fundamental biological function that has huge implications in the downstream process so this region would be likely conserved. The authors should find examples of not just having mutations in splicing regions but splicing mutations in sex-determining genes to be relevant and set reasonable hypotheses/expectations.

***Reply*** *: Example of splicing mutations in sex determining genes were added in the introduction. Please, see lines 135-138.*

**#Reviewer 2.** *Table 1 – Since the study examined the allele frequency between males and females, Table 1 should provide the number of male and female sequences used for the analysis.*

***Reply*** *: The number of male and female sequences used for the analysis were provided in Table 1.*

**#Reviewer 2**. *Some sites of the Ag1000 data only contain females so generalizing the sex-dependent patterns with skewed data can be misleading.*

***Reply*** *: We thank the authors for his point of view. Our objective was to look for any sex-dependent patterns. Since we did not find any, we didn’t generalize. We looked at the whole Ag1000 data for Anopheles gambiae (all countries). According to our hypothesis, if there was any sex-dependent pattern present, it should be observed at least in the sites (countries) with both female and male data. Furthermore, we were not expecting the sex-dependent pattern to be country-specific.*

**#Reviewer 2**. “Only Anopheles gambiae samples were considered in our study.” -> Anopheles gambiae s.s. (S form) only? Since the species gambiae and coluzzii were separated only in 2014, I think it would be best to clarify what you included for the readers.

***Reply*** *: In this study we used only Anopheles gambiae s.s. Details were added in the method section. Please, see line 151.*

**#Reviewer 2. **The first paragraph of the results section should be in the introduction. Also, readers who are not familiar with the sex determination process would wonder why sex determining gene is located on an autosome, not a sex chromosome. To educate readers, the authors should briefly describe how doublesex on autosome is involved in sex determination of Anopheles gambiae based on currently published literature in the introduction.

***Reply*** *: The first paragraph of the results section has been moved to the introduction. Please, see lines 105-113 in the revised manuscript.*

*Details on how doublesex is involved in sex determination of Anopheles gambiae was addressed in the revised manuscript. Please, see lines 91-97.*

**#Reviewer 2. **Figure 1. It would be best if female and male from the same location is side by side for a direct comparison. Males and females separate with different y-axis scales makes it difficult to compare. FR and GA colors are too similar.

***Reply*** *: The figure 1 was changed and became figure 2 with males and females separately with the same y-axis scales.*

**#Reviewer 2**. “The An. gambiae doublesex (Agdsx) gene is a candidate gene of interest for SIT, as a candidate for genetic modifications”-> candidate is repeated twice. Perhaps change it to “… is a candidate gene for genetic SIT strategy” or something to that effect?

 Important paper on this topic like https://www.ncbi.nlm.nih.gov/pmc/articles/PMC7955153/1 is not cited in this paper.

***Reply*** *: The sentence was corrected and the paper suggested was cited. Please see line 203.*

**#Reviewer 2**. “According to the D. melanogaster model18, the alternative splicing of Agdsx gene is governed by exon 5 skipping in male mosquitoes16 suggesting a silencing mechanism of the female-specific splice sites recognition (intron 4 acceptor and exon 5 donor sites) by the splicing machinery in males.” -> I think there is a new paper on this topic from Anopheles gambiae which sequenced female and male transcriptome so the author doesn’t have to deduce from the Drosophila sex termination model. Please check the latest papers by Krzywinski for updated literature on Anopheles gambiae sex determination.

***Reply*** *: Done*

**#Reviewer 2** “They appeared only is two mosquito populations (Burkina Faso and Cameroon) over the eight populations considered.” -> Burkina Faso and Cameroon are far apart so I wouldn’t minimize how widespread this mutation is based on the number of countries examined (2 out of 8 is still not a low number).

***Reply*** *: We thank the reviewer for this notice. However, they appeared in very few individuals, less than 1% in females and no more than 2% in males.*

# Peer review: Reviewer response for version 1

**DOI:** 10.21956/wellcomeopenres.19431.r48830

**Published:** 2022-03-11

**Authors:** Tony Nolan

**Affiliations:** 1 Department of Vector Biology, Liverpool School of Tropical Medicine, Liverpool, UK

The report looks at variations around the splicing sites in the gene doublesex, a transcription factor essential in determining male and female sexual dimorphism. It mines data from the AG1000g initiative, which is sampling wild caught *Anopheles* malaria vectors and sequencing their genomes. The approach taken here, to mine the same data and look for insight on genetic variation that would be informative in genetic control approaches, is welcome. The methods and approach taken are generally well described and background information provided on doublesex gives good context. Unfortunately, though, I think there are some issues with the rationale applied and these currently, in my view, would preclude the indexing of this study. These issues are detailed below but I do think the methodology applied if given a different focus – for example in focusing the analysis on target sites around the female-specific splice junction that are used in a specific form of genetic control called gene drive. These are detailed below:

**Major Points:**

- I’m struggling with the logic that SNPs in a splice site could definitively determine, and be sufficient for, sex-specific splicing. By extension, the fact that the SNPs are found in both sexes is taken to mean that these particular SNPs cannot be critical for alternative, sex-specific splicing. But all genes, and alleles thereof, must be found in males and females at some point unless linked to a sex determining chromosome or locus. Am I missing something? In short, I just don’t see how gender-specific SNPs will be helpful or informative (or at all likely) in this instance. The good thing at least is that your results confirm this.
- Gene drives that target the doublesex gene, and specifically, the female-specific exon, recognise and cleave target sites that are uniquely within the female-specific exon. In the first published example by Kyrou *et al.* in 2018 the target site overlaps the intron4-exon5 boundary (the region of study here). Therefore, a refocusing of the analysis approach used in the current report, but instead to the probability of variants occurring (and being tolerated) that might show positive selection (or not) in the face of a gene drive suppressing the population by targeting this region, would be a way to resolve the current limitations. This obviously would require a re-focusing of the manuscript and a tweaking of the analysis by the authors.
- Another potential approach (unrelated) is to look at the conservation across the introns in general, and how (as I guess it is) this conservation is higher towards the donor and acceptor splice sites (since these are more functionally constrained). This analysis might reveal the extent and nature of the splicing sites/ 

**Minor points:**

-  the *doublesex* ( *dsx*) gene, encoding somatic sexually dimorphic traits – I would tweak this language slightly: dsx does not encode these traits directly
- It is not explicitly stated (yet probably should be) that alternative, sex-specific splicing of dsx is critical for the initiation of downstream transcriptional pathways that underpin sexual dimorphism.
- “Y-chromosome shredding gene drive” could be read ambiguously – the gene drive *is* on the Y chromosome but shreds the X chromosome.
- Though the paragraph leads with ‘vector control’ in general a reader might mistakenly think that Wolbachia or RIDL strains have been developed for malaria vectors (rather than Aedes vectors of Dengue etc.).
- I would add the qualifier ‘local’ to elimination
- “Unfortunately, *An. gambiae dsx* gene ( *Agdsx*) has a different structure suggesting that *Agdsx* sex-specific splicing event is caused by a mechanism different from that of the *D. melanogaster dsx” I am not quite sure what is meant by this statement – from what I know the overall structure in terms of sequence conservation and splicing structure, as well as last coding exon being sex-specific etc is pretty well conserved between Anopheles and Drosophila.*
- “The SNPs association to the sex phenotype (male or female) was evaluated by running the association analysis using the general linear model (GLM) function in TASSEL.” – see my earlier comment. I think this approach needs rethinking.
- “The difference between the two sex-specific transcripts is due to the alternative splicing of exon 5. The latter is a cassette exon, which is retained in female and skipped in male transcript. The whole sequence of the female-specific exon 5 is included in the male intron 4 region and is spliced out. This gene structure causes a shift in intron/exon number in male. Thus, although male and female share the same exon/intron or intron/exon boundaries, they have common and specific splice sites.” – this would surely benefit from a figure (even if it’s been done before, it would help here in visualisation)
- “ *Agdsx* gene, 17,196 polymorphic sites were identified.” What is the definition for polymorphic here? A site where at least one individual in the sampled population that has a variant base compared to the published consensus genome?
- “Wherever both male and female mosquitoes are present (in Burkina Faso, Cameroon and Mayotte), the nucleotide diversity is similar between both sexes ( Figure 1). This was expected as male and female in each country make up a single population. In addition, no difference in the nucleotide diversity was observed between male populations from the three countries (Burkina Faso, Cameroon and Mayotte) ( Figure 1, top panel). The same trend was observed between female populations as well (Burkina Faso, Cameroon, Mayotte, Gabon, Ghana, Guinea, Equatorial Guinea and Uganda)” again, see earlier comment about rationale for looking at sex-specific differences.
- Figure 2 is nice.
- Figure 3 shows a heatmap of SNPs around exon 5 donor site, but the corresponding heatmap for the region around intrn4/exon 5 (for which a nice cartoon was made in Fig 2) is not shown.
- Re: the ‘absence’ of transformer in *An. gambiae* – there are several competing hypotheses for this, and not all are mentioned here (e.g difficulty with finding it by homology-based approaches due to extremely rapid sequence divergence)
- Throughout the article, it’s never really made explicitly clear *how* this information on Dsx, and the splicing details specifically, would be relevant for genetic control, and SIT (which is mentioned frequently) in particular.

Is the work clearly and accurately presented and does it cite the current literature?

Yes

If applicable, is the statistical analysis and its interpretation appropriate?

Partly

Are all the source data underlying the results available to ensure full reproducibility?

Yes

Is the study design appropriate and is the work technically sound?

Partly

Are the conclusions drawn adequately supported by the results?

No

Are sufficient details of methods and analysis provided to allow replication by others?

Yes

Reviewer Expertise:

vector control, gene drive, genetic control, molecular biology

I confirm that I have read this submission and believe that I have an appropriate level of expertise to confirm that it is of an acceptable scientific standard, however I have significant reservations, as outlined above.

# Response

**Published:** 2022-12-03

**Authors:** Oswald Djihinto

**Affiliations:** Tropical Infectious Diseases Research Centre (TIDRC), University of Abomey-Calavi, Benin

**#Reviewer 1. **The report looks at variations around the splicing sites in the gene doublesex, a transcription factor essential in determining male and female sexual dimorphism. It mines data from the AG1000g initiative, which is sampling wild caught Anopheles malaria vectors and sequencing their genomes. The approach taken here, to mine the same data and look for insight on genetic variation that would be informative in genetic control approaches, is welcome. The methods and approach taken are generally well described and background information provided on doublesex gives good context. Unfortunately, though, I think there are some issues with the rationale applied and these currently, in my view, would preclude the indexing of this study. These issues are detailed below but I do think the methodology applied if given a different focus – for example in focusing the analysis on target sites around the female-specific splice junction that are used in a specific form of genetic control called gene drive.

***Reply*** *:* *We thank the reviewer for suggesting a new orientation for the work. However, analysis of target sites around the female-specific splice junction used for the genetic control called gene drive has already been done by Kyrou et al. (2018). Despite using cage experiments over 4 mosquito generations, the authors revealed low-frequency indels at the target site.*

*Kyrou K, Hammond AM, Galizi R, Kranjc N, Burt A, Beaghton AK, et al. A CRISPR–Cas9 gene drive targeting doublesex causes complete population suppression in caged Anopheles gambiae mosquitoes. Nature Biotechnology. 2018;36: 1062–1066. doi:10.1038/nbt.4245*

**#Reviewer 1. **I’m struggling with the logic that SNPs in a splice site could definitively determine, and be sufficient for, sex-specific splicing. By extension, the fact that the SNPs are found in both sexes is taken to mean that these particular SNPs cannot be critical for alternative, sex-specific splicing. But all genes, and alleles thereof, must be found in males and females at some point unless linked to a sex determining chromosome or locus. Am I missing something? In short, I just don’t see how gender-specific SNPs will be helpful or informative (or at all likely) in this instance. The good thing at least is that your results confirm this.

***Reply*** *:* *The reviewer is correct. The presence of SNPs in both sexes means that these particular SNPs cannot be critical for alternative sex-specific splicing. However, this study was aiming to investigate any gender-specific SNP in the target splice site sequences that could be critical for exon 5 skipping splicing mechanism. This objective was driven by reports of SNPs in the splice site that lead to an alternative exon splicing.*

**#Reviewer 1. **Gene drives that target the doublesex gene, and specifically, the female-specific exon, recognise and cleave target sites that are uniquely within the female-specific exon. In the first published example by Kyrou et al. in 2018 the target site overlaps the intron4-exon5 boundary (the region of study here). Therefore, a refocusing of the analysis approach used in the current report, but instead to the probability of variants occurring (and being tolerated) that might show positive selection (or not) in the face of a gene drive suppressing the population by targeting this region, would be a way to resolve the current limitations. This obviously would require a re-focusing of the manuscript and a tweaking of the analysis by the authors.

***Reply*** *:* *We are grateful to the reviewer for this suggestion. However, a re-focusing of the manuscript to tackle the suggested aspect of the reviewer will lead to rewriting the whole manuscript content, which will not suit the title of the current manuscript. We will address the minor comments in this revision of the manuscript and later on, submit another manuscript on the investigation of a likely positive selection in the female-specific exon* *.*

**#Reviewer 1. **Another potential approach (unrelated) is to look at the conservation across the introns in general, and how (as I guess it is) this conservation is higher towards the donor and acceptor splice sites (since these are more functionally constrained). This analysis might reveal the extent and nature of the splicing sites/

***Reply*** *: We thank the reviewer for this suggestion. We will consider it when investigating the* *likely positive selection in the female-specific exon.*

**#Reviewer 1.** The doublesex (dsx) gene, encoding somatic sexually dimorphic traits – I would tweak this language slightly: dsx does not encode these traits directly.

***Reply*** *: The sentence was rephrased as follow: “…* *the doublesex (dsx) gene, involved in mosquitos’ somatic sexually dimorphic traits determination.” Please, see lines 18 and 19.*

**#Reviewer 1.** “Y-chromosome shredding gene drive” could be read ambiguously – the gene drive is on the Y chromosome but shreds the X chromosome.

***Reply*** *: Explanation was made as follow: “Y-chromosome shredding gene drive (the gene drive is on the Y chromosome but shreds the X chromosome)”. Please, see line 70 and 71.*

**#Reviewer 1.** Though the paragraph leads with ‘vector control’ in general a reader might mistakenly think that Wolbachia or RIDL strains have been developed for malaria vectors (rather than Aedes vectors of Dengue etc.).

***Reply*** *: The paragraph was corrected. But yes Wolbachia has been developed for malaria vectors. Please see lines 66-69*

**#Reviewer 1. **I would add the qualifier ‘local’ to elimination

***Reply*** *: Done*

**#Reviewer 1**. “Unfortunately, An. gambiae dsx gene (Agdsx) has a different structure suggesting that Agdsx sex-specific splicing event is caused by a mechanism different from that of the D. melanogaster dsx” I am not quite sure what is meant by this statement – from what I know the overall structure in terms of sequence conservation and splicing structure, as well as last coding exon being sex-specific etc is pretty well conserved between *Anopheles *and *Drosophila*.

***Reply*** *: The structure that we are talking about here is the gene organization and regulatory elements in D. melanogaster dsx (Dmdsx) and Agdsx. Indeed, Scali et al., (2005) have reported that D. melanogaster female dsx gene has 4 exons while An. gambiae (Agdsx) has 7 exons. The exon 4 is splice out in female Dmdsx transcript while the exon 5 is the cassette exon in Agdsx.* *In *Dmdsx*, the inclusion or the exclusion of the female-specific exon depends on the presence of a weak 3’ splice site in the preceding intron. Genomic sequence and transcript analysis indicate that in *An. gambiae*, splicing into sex-specific transcripts does not depend on the choice of an alternative 3’ splice site (Scali et al., 2005). The 3’ acceptor site preceding exon 5 does not appear to be weak. Although regulatory elements (splice enhancers and potential purine-rich enhancers) are found in the 3’ UTR of exon 5 in Agdsx, these elements are found much further downstream from the 3′ splice acceptor site than in *Drosophila*, immediately upstream of the 5′ donor site of intron 5.*

*Scali C, Catteruccia F, Li Q, Crisanti A. Identification of sex-specific transcripts of the Anopheles gambiae doublesex gene. J Exp Biol. 2005;208: 3701–3709. doi:10.1242/jeb.01819*

**#Reviewer 1.** “The SNPs association to the sex phenotype (male or female) was evaluated by running the association analysis using the general linear model (GLM) function in TASSEL.” – see my earlier comment. I think this approach needs rethinking.

***Reply*** *: This section was removed from the revised manuscript.*

**#Reviewer 1. **“The difference between the two sex-specific transcripts is due to the alternative splicing of exon 5. The latter is a cassette exon, which is retained in female and skipped in male transcript. The whole sequence of the female-specific exon 5 is included in the male intron 4 region and is spliced out. This gene structure causes a shift in intron/exon number in male. Thus, although male and female share the same exon/intron or intron/exon boundaries, they have common and specific splice sites.” – this would surely benefit from a figure (even if it’s been done before, it would help here in visualisation).

***Reply*** *: A figure was proposed. Please see Figure 1 (line 113*) *. This came in the introduction section according to the reviewer 2.*

**#Reviewer 1** “ Agdsx gene, 17,196 polymorphic sites were identified.” What is the definition for polymorphic here? A site where at least one individual in the sampled population that has a variant base compared to the published consensus genome?

***Reply*** *: Yes, the polymorphic sites are nucleotide position where at least one individual in the sampled population has a variant base compared to the published AGAMP4 consensus genome.*

**#Reviewer 1**. “Wherever both male and female mosquitoes are present (in Burkina Faso, Cameroon and Mayotte), the nucleotide diversity is similar between both sexes (Figure 1). This was expected as male and female in each country make up a single population. In addition, no difference in the nucleotide diversity was observed between male populations from the three countries (Burkina Faso, Cameroon and Mayotte) (Figure 1, top panel). The same trend was observed between female populations as well (Burkina Faso, Cameroon, Mayotte, Gabon, Ghana, Guinea, Equatorial Guinea and Uganda)” again, see earlier comment about rationale for looking at sex-specific differences.

***Reply*** *: Here we were not looking at sex-specific differences. Instead, we reported the genetic diversity of the dsx gene between mosquito populations. We split the data in female and male because all countries did not have both sex.*

**#Reviewer 1. **Figure 3 shows a heatmap of SNPs around exon 5 donor site, but the corresponding heatmap for the region around intrn4/exon 5 (for which a nice cartoon was made in Fig 2) is not shown.

***Reply*** *: The heatmap for the region around intron4/exon 5 was not shown because n* *o SNP was neither found in the female intron 4 splice acceptor site or in the corresponding region in male.*

**#Reviewer 1. **Re: the ‘absence’ of transformer in An. gambiae – there are several competing hypotheses for this, and not all are mentioned here (e.g difficulty with finding it by homology-based approaches due to extremely rapid sequence divergence)

***Reply*** *: We thank the reviewer for this input. This statement was removed from the revised manuscript.*

**#Reviewer 1. **Throughout the article, it’s never really made explicitly clear how this information on Dsx, and the splicing details specifically, would be relevant for genetic control, and SIT (which is mentioned frequently) in particular.

***Reply*** *: In this manuscript, we highlight that dsx is a promising target for genetic control and SIT. For this reason, we assumed that it is important to understand the mechanisms underlying it alternative splicing in the targeted vectors since the only well-known model comes from D. melanogaster. In An. gambiae, there are still some gap in all components involved in the dsx splicing. The identification of the remaining factors could lead to the characterization of novel genetic and biochemical targets for inducing genetic sterility*
